# Towards Efficient Lignin Removal: A Box–Behnken Design Approach to Corncob Delignification Using Deep Eutectic Solvents

**DOI:** 10.3390/molecules31183202

**Published:** 2026-09-11

**Authors:** Rebeca Astorga-Trejo, Luis Ramiro Caso-Vargas, Efraín Rubio-Rosas, Leslie Susana Arcila-Lozano, María Guadalupe Aguilar-Uscanga, Claudia Castro-Martínez

**Affiliations:** 1 Laboratorio de Bioenergéticos, Centro Interdisciplinario de Investigación para el Desarrollo Integral Regional-Unidad Sinaloa, Instituto Politécnico Nacional, Boulevard Juan de Dios Bátiz Paredes 250, Guasave 81101, Sinaloa, Mexico; rastorgat2000@alumno.ipn.mx; 2 Facultad de Ciencias Biológicas, Benemérita Universidad Autónoma de Puebla, Puebla 72570, Puebla, Mexico; ramiro.caso@correo.buap.mx; 3 Dirección de Innovación y Transferencia del Conocimiento, Centro Universitario de Vinculación y Transferencia de Tecnología, Benemérita Universidad Autónoma de Puebla, Puebla 72592, Puebla, Mexico; e.rubio.rosas@gmail.com; 4 Centro de Investigación en Biotecnología Aplicada (CIBA Tlaxcala), Instituto Politécnico Nacional, Investigadoras e Investigadores por México, Secretaría de Ciencia, Humanidades, Tecnología e Innovación, Ex-Hacienda San Juan Molino, Carretera Estatal Manzana Tepetitla Km 1.5, Santa Inés Tecuexcomac 90700, Tlaxcala, Mexico; larcila@secihti.mx; 5 Laboratorio de Bioingeniería y Planta Piloto, Instituto Tecnológico de Veracruz, Av. Miguel Ángel de Quevedo 2779, Formando Hogar, Veracruz 91897, Veracruz, Mexico; maria.au@veracruz.tecnm.mx

**Keywords:** biomass, corncob, delignification, deep eutectic solvent

## Abstract

Efficient pretreatment is essential for overcoming lignocellulosic biomass recalcitrance and enabling its use in biotechnological processes. In this study, corncob, an agricultural by-product rich in cellulose and hemicellulose, was subjected to acid pretreatment to remove hemicellulose and subsequently delignified using two deep eutectic solvent (DES) systems: choline chloride:urea and choline chloride:glycerol. The effect of temperature and reaction time was evaluated for both systems; ChCl:U was selected for optimization due to its superior lignin removal performance under the conditions evaluated. Temperature, time, and solid–liquid ratio (SLR) were optimized using a Box–Behnken design to maximize lignin removal. Under optimal conditions (113 °C, 5.7 h, 1:16.2 g/mL), 59.086% lignin removal was achieved. Temperature and reaction time significantly influenced delignification, whereas the solid–liquid ratio had a minimal effect. Structural changes in biomass throughout the process were characterized by compositional analysis, Fourier transform infrared spectroscopy (FTIR), and scanning electron microscopy (SEM), confirming effective delignification. To the best of our knowledge, there are few studies that comprehensively characterize DES-treated corncob using FTIR and SEM analysis. These findings demonstrate that choline chloride:urea has potential as a sustainable and effective pretreatment system for lignocellulosic biorefineries based on agricultural residues.

## 1. Introduction

The raw material utilized during the biotechnological production of a compound of commercial interest can account for up to 80% of the total production cost of a metabolite (bioethanol, xylitol, biohydrogen, and enzymes, among others) [1]. One alternative to reduce these costs (by 30–40%) is the use of lignocellulosic biomass (LB). Agricultural residues represent one of the most abundant types of biomasses in nature and are mainly composed of cellulose (35–50%), hemicellulose (22–32%), and lignin (10–25%). Lignocellulosic biomass has a complex and recalcitrant structure that limits its utilization in biotechnological processes. Therefore, pretreatment strategies are required to modify its structure to obtain smaller bioavailable molecules, such as fermentable sugars (glucose, xylose, arabinose) and/or aromatic compounds for use as raw materials [2].

Globally, Mexico is one of the leading corn producers. In 2024, production reached 21.49 million tons [3]. The by-products of corn grain harvesting include the stalk, leaves, and corncob. Corncob is generated in abundant quantities (approximately 20–30% of corn production) during the process of separating the corn grain from the cob. It has traditionally been used as livestock forage, fertilizer for farmland, or burned in open air, contributing to soil erosion and environmental pollution [4,5]. Due to its high availability and low cost, corncob could function as a raw material to produce bioenergy, enzymes, and sweeteners through various fermentative routes [6].

Deep eutectic solvents have emerged as an alternative to conventional delignification solvents. DESs are liquid complexes typically made up of two or three components that can bond together to create a eutectic mixture with a melting point lower than that of each individual constituent [7]. Moreover, given that most immiscible solid-state mixtures have a eutectic point, the eutectic temperature must be lower than that of an ideal eutectic mixture [8]. DESs are generally composed of a hydrogen bond acceptor (HBA) and a hydrogen bond donor (HBD). Common HBAs are either ammonium or phosphonium halides. Choline chloride (ChCl) is frequently employed due to its low cost and low toxicity [9]. HBDs can be selected from various types of substances such as amines, carbohydrates, carboxylic acids, and alcohols [10,11]. DESs are considered inexpensive, easy-to-prepare compounds that are biodegradable, biocompatible, and renewable [12]. Affordability, non-toxicity, low viscosities and melting points, as well as potential for recycling, are amongst DESs’ advantages for technological applications [13].

The results obtained by different research groups on the use of DESs during the pretreatment stage of lignocellulosic biomass highlight the capacity of these compounds to break down lignin and xylan molecules and reduce the degree of cellulose polymerization [14]. Acidic DESs based on choline chloride and organic acids are known for exhibiting high lignin solubility [15]; nevertheless, this acidified medium could catalyze a chain reaction that turns xylan into xylose and further converts xylose into furfural [16]. Kohli et al. [17] pretreated wood residues using choline chloride and organic acid-based DESs, achieving varying lignin removal percentages (20–70%).

On the other hand, basic DESs have been proposed to treat biomass effectively while preserving hemicellulose [18]. Zhao et al. [19] studied the capacity of six basic DES to remove lignin from wheat straw and found that systems with stronger basicity removed lignin more efficiently; lignin removal ranged from 3.4% using a ChCl:glycerol system (pH 7.7) to 81% using a ChCl:monoethanolamine (pH 10.9) system.

Several researchers have emphasized the need for more in-depth studies due to significant variations in lignin removal percentages. These variations are linked to the process operating conditions, including raw material characteristics (stage of maturity, source), the type of DES system applied, and physicochemical variables (temperature, time, agitation, and solid–liquid ratio) [20,21].

The aim of this study was to evaluate the effect of physicochemical variables on lignin removal from corncobs using choline chloride:urea and choline chloride:glycerol systems, to maximize delignification by optimizing process variables, and to characterize biomass structural changes induced by the treatment using compositional analysis, Fourier transform infrared spectroscopy and scanning electron microscopy.

## 2. Results and Discussion

### 2.1. Biomass Compositional Analysis

Compositional analysis was performed on raw biomass, on the biomass after acid hydrolysis, and after treatment with DESs. The composition of raw corncob was determined to be 34.59 ± 2.56% glucan, 33.60 ± 1.95% xylan, and 20.193 ± 0.13% lignin. Biomass was then treated with dilute sulfuric acid, after which most of the xylan content was removed and the lignin content was 30.59 ± 0.912% (Supplementary Table S1).

Although there are differences in corncob composition, the results obtained in this work are consistent with the ranges found in the literature. Fialho et al. [22] reported glucan and xylan contents similar to our findings (35.74% and 33.32%, respectively); however, the lignin content of their biomass (14.57%) was lower than that observed in this study. On the other hand, Du reported an 18.97% lignin content, but lower cellulose (29.30%) and hemicellulose (27.50%) proportions. Interestingly, Ponce-Fernández et al. [23] characterized corn residues collected from the same region as the present work, and found that corncob composition was 30.92% cellulose, 45.9% hemicellulose, and 15.68% lignin. Structural changes in biomass are expected even when using the same plant species; authors agree that lignocellulosic composition may vary depending on factors such as origin, climate conditions, soil composition, and fertilizer use [24,25].

### 2.2. Temperature and Time Effect on Lignin Removal

Although the precise mechanisms behind lignin fractionation and solubility are not yet fully understood [26], it is believed that the type of DES used significantly influences delignification efficiency, while reaction parameters such as time and temperature have a significant effect on biomass pretreatment [6,27]. In this study, the effect of these parameters was evaluated for two DES systems: choline chloride with urea (ChCl:U) and choline chloride with glycerol (ChCl:Gly). This section evaluates the impact of these variables on pretreatment and provides comparative information on the performance of the two DES systems.

**Figure 1:**
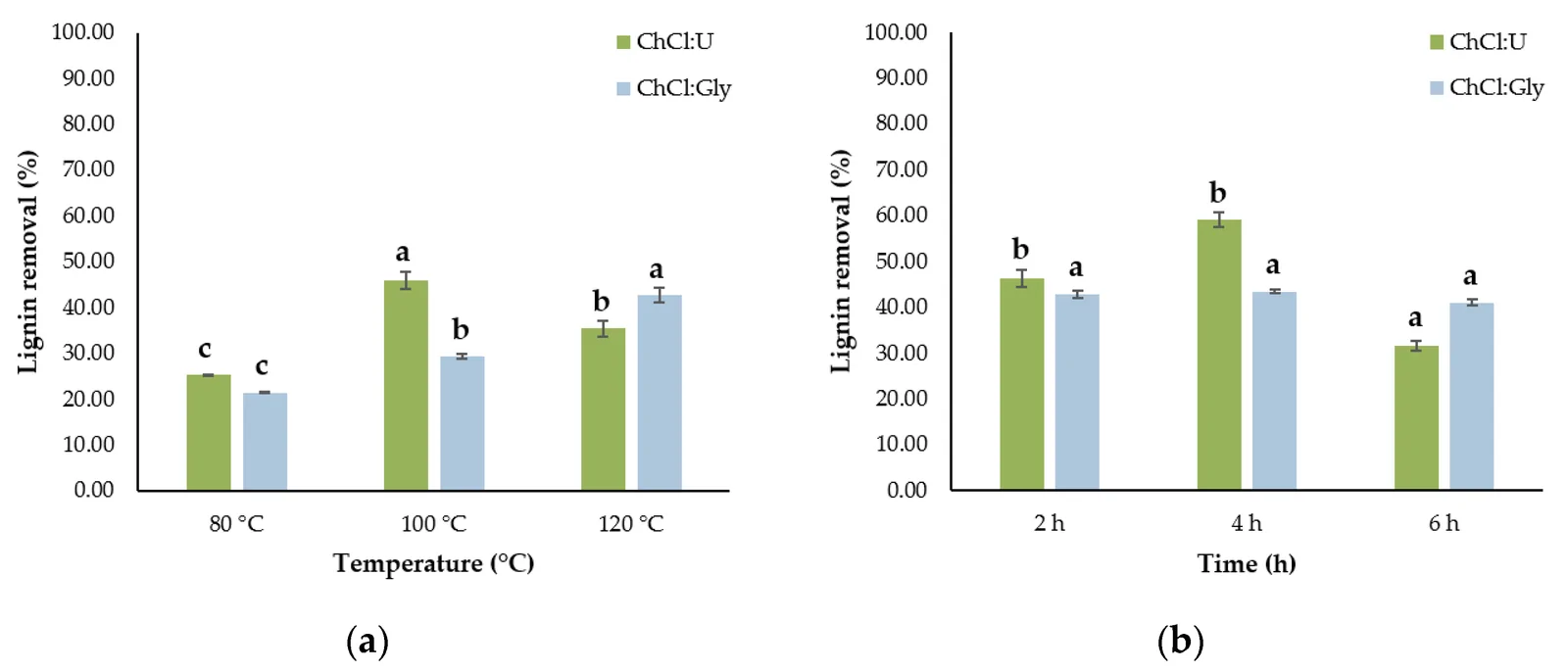
Effect of (**a**) temperature (2 h treatment) and (**b**) time (at 100 °C) on lignin removal using DESs. Values represent the mean ± standard deviation (SD) of three independent experiments (*n* = 3). Different lowercase letters indicate statistically significant differences among treatments according to a one-way ANOVA followed by Tukey’s HSD test (*p* < 0.05).

The effect of temperature on the treatment of corncob is presented in Figure 1a, where different responses were observed depending on the DES composition. In the ChCl:Gly system, lignin removal increased progressively with temperature, reaching a 42.73% lignin removal at 120 °C. In contrast, the urea system shows a non-linear response to temperature; a maximum lignin removal of 45.98% was achieved at 100 °C, followed by a decline as temperatures increased to 120 °C.

The positive effect of increasing temperature can be attributed to the reduction in DES viscosity, which enhances solvent interaction with lignin–carbohydrate linkages and improves lignin solubilization [28]. However, the decrease in lignin removal for the urea system at 120 °C may be associated with the thermal stability of the solvent. Marchel et al. [29] reported that ChCl:urea decomposes when subjected to prolonged treatments at temperatures above 100 °C, reducing its delignification efficiency.

Figure 1b illustrates the effect of time on lignin removal on both DES systems. Time had a greater effect on lignin removal when using urea as HBD. The highest removal (58.98%) using this system was achieved after 4 h of treatment, followed by a decline at 6 h. Meanwhile, lignin removal in the ChCl:Gly system was around 40% regardless of treatment time. Even though increasing pretreatment time generally enhances lignin solubilization by allowing for longer interactions between DES and the lignocellulosic matrix, extended treatment time could cause degradation or condensation that limits lignin extraction, as observed by Dandash et al. [30].

Using a combination of choline chloride, lactic acid, and formic acid, Oh et al. [31] obtained a lignin removal of 72% and found that delignification increased with temperature ranging from 80 °C to 130 °C. Nevertheless, no further lignin removal was observed when the pretreatment temperature was increased to 150 °C. The lignin removal rate increased with increasing time up to 6 h, only to reach equilibrium in longer treatments.

Similarly, Zhu et al. [32] treated rice straw with ChCl:urea at different temperatures and reaction times and discovered that, in that particular biomass, temperature played a more important role in the removal of lignin. Experiments showed that increasing the temperature allowed a greater percentage of lignin to be removed, while increasing time increments had no effect on the treatment.

During this evaluation, the ChCl:U system showed a clear response to variations in temperature and time, whereas a limited response to reaction time was observed in the glycerol system, suggesting a low sensitivity to the operating conditions evaluated. Although lignin removal tended to increase with temperature, the operational range explored in this study was limited by the maximum temperature attainable with the available experimental setup (Supplementary Table S2 and Figure S1). Consequently, further optimization efforts were focused on the ChCl:U system.

According to Xu et al. [21], in addition to temperature and reaction time, the solid–liquid ratio is a key process variable to achieve high lignin removal. Moreover, the use of optimization methods has revealed that the biomass and specific DES studied respond differently to reaction conditions [33]. Based on these findings, temperature (80 to 120 °C), time (2 to 8 h), and the solid–liquid ratio (1:10 to 1:20 g:mL) were evaluated using response surface methodology to determine the optimal conditions for lignin removal on the ChCl:U system.

### 2.3. Box–Behnken Design Statistical Analysis

The optimization of lignin removal from corncob using a ChCl:U DES system was investigated through a Box–Behnken design (BBD); the experimental design matrix and corresponding responses are provided in Table S3. A quadratic model best described the experimental data, as presented in Equation (1).

(1) $$\begin{array}{ll} Lignin\;removal\;\left(\%\right) & \\ & =-154.77458+2.15309\times Temperature+13.89528\times Time+6.42192\times SLR\\ & -0.063689\times Temperature\times Time-0.010017\times Temperature\times SLR\\ & +0.085760\times Time\times SLR-0.007198\times {Temperature}^{2}-0.708605\times {Time}^{2}\\ & -0.178220\times {SLR}^{2} \end{array}$$

The ANOVA results for lignin removal are displayed in Table 1. The model was significant with a *p*-value of 0.043; temperature (A) and time (B) had significant effects on lignin removal, with *p*-values of 0.0185 and 0.0150, respectively. In contrast, the solid–liquid ratio (C) had no significant effect on the treatment. Interestingly, the quadratic term B^2^ (*p* = 0.0287) was significant to the model, meaning that the effect of time on lignin removal is non-linear and that beyond a certain point, increasing or decreasing the reaction time may lead to diminished results.

**Figure 2:**
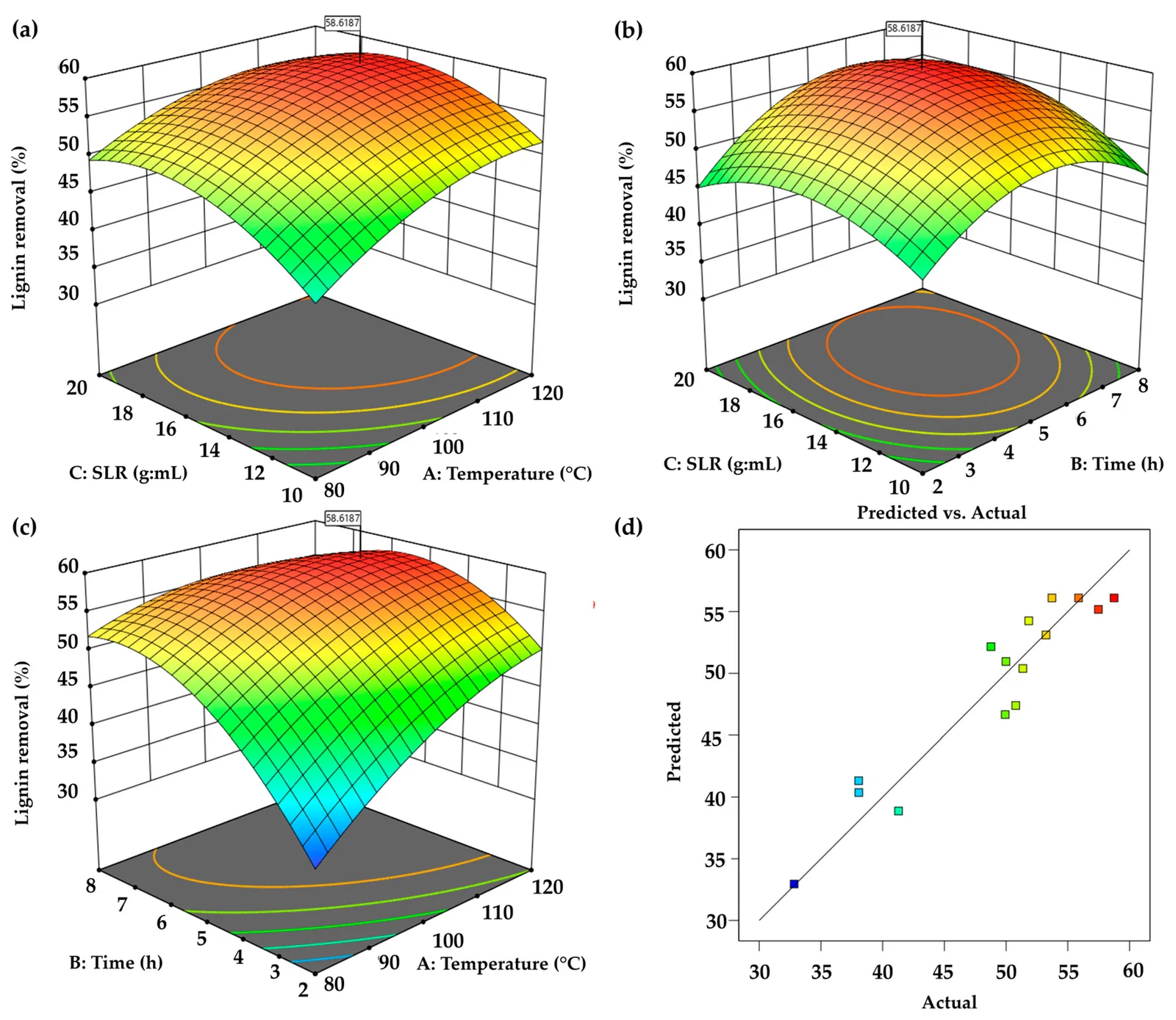
ChCl:U lignin removal surface response graphs. (**a**) Interaction between reaction time and SLR, (**b**) interaction between reaction temperature and SLR, (**c**) interaction between reaction time and temperature, (**d**) predicted vs. actual values. Color gradient represents lignin removal (%), with blue corresponding to lower values and red to higher values.

This is noticeable in Figure 2a, where it is shown that lignin removal decreased when reaction times exceed 6 h, possibly due to saturation or degradation effects. Figure 2b shows that higher temperatures significantly improved lignin elimination, while SLR effects were less pronounced. Figure 2c shows the interaction between temperature and time and reveals that lignin removal peaked at elevated temperatures and intermediate reaction times.

The coefficient of determination (R^2^) of the model was 0.902, with an adjusted R^2^ of 0.72, indicating that 72% of the variability in lignin removal is explained by the model, accounting for the number of terms included. Due to the difference between R^2^ and adjusted R^2^, our model is used only as a representation of the experimental response within the studied space. Residual plots can be found in Supplementary Figure S2. As can be seen in Figure 2d, experimental data and predicted values display reasonable agreement, despite some deviations.

Using the desirability function, the optimal conditions for maximizing lignin removal were identified; the software predicted a lignin removal percentage of 58.62% under the conditions of 113 °C, 5.7 h, and a solid–liquid ratio of 1:16.2. To validate the model, experiments were conducted in triplicate under the predicted optimal conditions. The experimental results for lignin removal were 59.086% with a standard deviation of 3.64; this demonstrates good agreement with the model-predicted results and those obtained experimentally.

Costa-Trigo et al. [34] investigated the effects of parameters such as time, reaction temperature, and the solid–liquid ratio on the delignification of chestnut bars using choline chloride and urea. The highest percentage of lignin removal (approximately 40%) was achieved in a treatment at 100 °C, for 16 h and with an SLR of 1:20. The authors also highlighted that temperature has a greater effect on lignin removal at short reaction times rather than long treatments.

On the other hand, Chia et al. [35] applied choline chloride-based DES with lactic acid as HBD to treat palm oil industry residues and achieved 53.6% delignification (similar to this study) on a 2.7 h treatment at 126 °C and an SLR of 1:10. The authors reached the conclusion that high temperatures and high substrate loadings allow for greater lignin removals even at moderate times. In contrast, in this study, lignin removal peaked at high temperatures and intermediate reaction times, while solid loading had a lesser impact.

Similar to our results, in a study performed on corncob using ChCl:urea, approximately 58% of lignin was removed in a treatment at 130 °C, with a solid-to-liquid ratio of 1:10 and a duration of 2 h. It was concluded that higher temperatures promote lignin removal; however, the combined effect of temperature and reaction time was not investigated [13].

### 2.4. FTIR Analysis of Raw Corncob, Sulfuric Acid-Treated Corncob and ChCl:U-Treated Corncob

**Figure 3:**
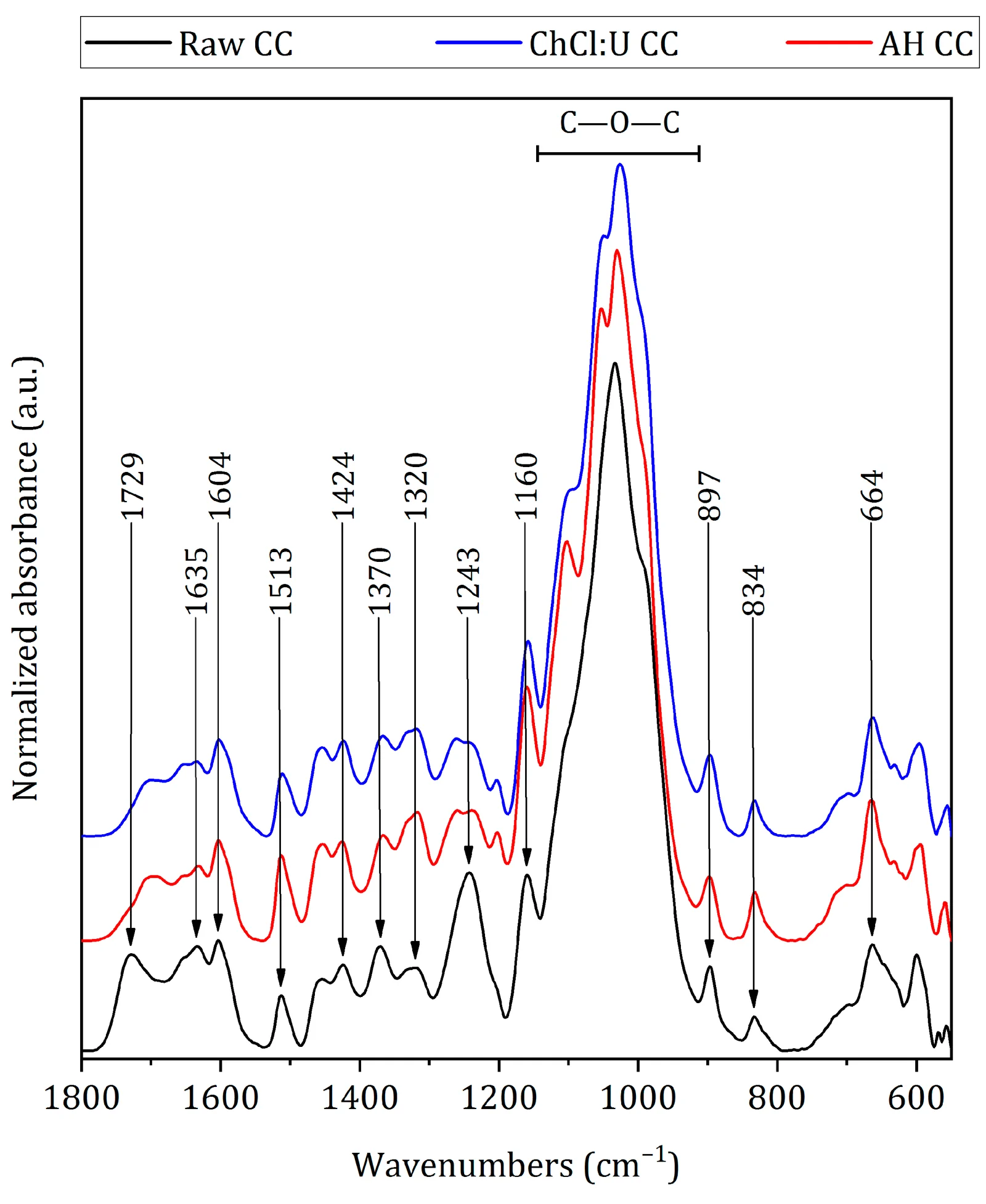
Vector-normalized ATR-FTIR spectra of raw corncob (raw CC), acid-hydrolyzed corncob (AH CC), and ChCl:U-treated corncob (ChCl:U CC).

FTIR analyses were carried out to examine the changes in biomass composition after treatments. Plant cell walls contain cellulose, hemicellulose, pectin, structural proteins, and lignin. Accordingly, several spectral features can be observed in the raw corncob spectrum within the 1800–550 cm^−1^ region. Most of these bands are also present after the AH and DES treatments, although changes in their relative intensities, and slight shifts in wavenumber and band shape can be observed (Figure 3). The assignments of the principal absorption bands discussed throughout this section are summarized in Table S4.

The band at 1729 cm^−1^ corresponds to the ν(C=O) vibration and is mainly attributed to ester carbonyl groups from acetylated hemicellulose, with possible contributions from ferulate and p-coumarate esters associated with arabinoxylan–lignin linkages, as well as protonated carboxyl groups from uronic acids. Pectins are present in corncob at concentrations as low as 1% [36]; therefore, the contribution of their uronic acids, mainly galacturonic acid, to the ν(C=O) band is expected to be minimal. Conversely, hemicelluloses account for a large proportion of the dry weight of corncobs, approximately 45% [23]. Arai et al. [37] reported that uronic acids represent 3.6% of the isolated corncob xylan fraction, which constitutes the main hemicellulosic polysaccharide fraction. They also determined that the main uronic acid in corncob xylan is 4-O-methyl-D-glucuronic acid. In addition, acetyl, feruloyl, and p-coumaroyl substituents were present at concentrations of 3.9%, 1.4%, and 0.4%, respectively.

The band at 1634 cm^−1^ overlaps several contributions, including δ(H—O—H) of adsorbed water, aromatic skeletal vibrations, mainly ν(C=C) of lignin, and conjugated carbonyl groups [38,39,40]. Consequently, tracking delignification in lignocellulosic substrates using only this band is not recommended due to potential interference from sample moisture and ambient water vapor. The sensitivity of this region to absorbed water in cellulose has been reported previously by Cichosz et al. [41], who found a strong relationship between cellulose moisture content and the intensity of the band located in the 1646–1636 cm^−1^ region (R^2^ = 92.6%; SEP = 0.7 wt.%). When the use of this band is unavoidable, it is critical to minimize these effects by storing the samples in a desiccator, acquiring spectra in a moisture-controlled room, and recording background scans frequently during the FTIR analysis of multiple samples. In our case, all these conditions were fulfilled. In the present study, the moisture content of the raw corncob samples was determined to be 4.26 ± 0.10%. This low variability helped establish a consistent band shape and intensity, thereby reducing the masking effect of adsorbed water and allowing the underlying molecular vibrations to dominate the band profile. In addition, previous studies have shown that atmospheric correction to ATR-FTIR effectively reduces interference from ambient water vapor [42].

In contrast to the band at 1634 cm^−1^, the absorptions at 1603 and 1513 cm^−1^ are located outside the main δ(H—O—H) contribution of adsorbed water and are more specifically associated with aromatic skeletal vibrations of lignin. The band at 1603 cm^−1^ is mainly attributed to ν(C=C) of the aromatic rings of p-hydroxyphenyl, guaiacyl, and syringyl units, all three of which are reported to be present in corn [37], whereas the band at 1513 cm^−1^ is one of the most characteristic lignin-associated absorptions and is related to aromatic skeletal vibrations of guaiacyl–syringyl units [43].

The band at 1424 cm^−1^ includes aromatic skeletal vibrations of lignin together with δ(CH_2_) contributions from cellulose. In isolated lignin, absorptions around 1420–1430 cm^−1^ are commonly assigned to aromatic ring vibrations and C—H bending [39,44]. Nevertheless, in whole lignocellulosic biomass, this region also contains an important contribution from CH_2_ bending of cellulose. Chen et al. [45], working with 200 maize germplasms, assigned the band near 1430 cm^−1^ to cellulose and found that its intensity was significantly related to hemicellulose content as well. The dual contribution of lignin and polysaccharides explains why this band is commonly used in the lateral order index, calculated as A_1430_/A_893_, to evaluate changes in cellulose organization [46]. Therefore, it should be considered as an indicator of general cell-wall modification rather than as an exclusive marker of delignification.

The band at 1370 cm^−1^ is primarily assigned to C—H deformation vibrations in cellulose and hemicellulose, including δ(CH) and δ(CH_2_) modes of the glucopyranose units [38]. In lignin, minor contributions from methyl and methoxy groups may also occur in this region [47]. In this case, as the spectra correspond to corncob samples rather than just isolated lignin, the polysaccharide signals are expected to dominate. Similarly, the band at 1320 cm^−1^ also contains overlapping contributions from lignin and cellulose. In polysaccharides, bands around 1315–1320 cm^−1^ are generally assigned to CH_2_ wagging vibrations of cellulose [6]. In isolated lignin, however, this region is commonly associated with aromatic C—O stretching of syringyl structures and condensed guaiacyl units [48].

The band at 1243 cm^−1^ corresponds to aromatic ν(C—O) in lignin, including aryl–ether and methoxy-containing structures and some contribution from hemicellulosic C—O vibrations. In isolated lignin, the closest characteristic absorption is often located between 1265 and 1270 cm^−1^ and is commonly related to guaiacyl structures [43].

Another composite signal is found in the band at 1160 cm^−1^. In cellulose and hemicellulose, it is mainly associated with asymmetric ν(C—O—C) vibrations of β-(1→4)-glycosidic linkages and with skeletal vibrations of the carbohydrate backbone. Chen et al. [45] assigned the band at 1163 cm^−1^ to asymmetric C—O—C stretching in cellulose and found that its intensity was significantly related to cellulose, hemicellulose, and holocellulose contents in maize; however, lignin may also contribute to this region. It is also noteworthy that absorptions between 1180 and 1140 cm^−1^ respond to moisture-induced changes as reported by Guo et al. [49], who observed that, with increasing humidity, the ~1160 cm^−1^ band in lignocellulosic material from *Ginkgo biloba* L. shifted towards lower wavenumbers, concluding that the adsorption of water affected the O(3)H⋯O(5) hydrogen bond of glucose monomers in cellulose.

The signal at 897 cm^−1^ is primarily a carbohydrate-associated absorption and is commonly assigned to C—H deformation at the anomeric carbon and to vibrations of β-(1→4)-glycosidic linkages in cellulose and hemicellulose [50].

Finally, the band at 833 cm^−1^ arises from the out-of-plane aromatic C—H deformation in substituted lignin rings [51]. The position of the out-of-plane C—H deformation depends strongly on the substitution pattern of the aromatic ring and on contributions from hydroxycinnamates. Consequently, the band assignment in this low-wavenumber region varies among lignins. This is especially relevant in grasses such as maize, where p-coumarate and ferulate moieties are integral components of lignin and lignin–arabinoxylan networks [52]. For this reason, the band at 833 cm^−1^ should be regarded as an aromatic lignin-associated signal rather than as an exclusive marker of a single lignin subunit. A decrease in its intensity, particularly when accompanied by reductions at 1603, 1513, 1320, and 1243 cm^−1^, would support the removal or structural modification in aromatic lignin components during treatment.

**Figure 4:**
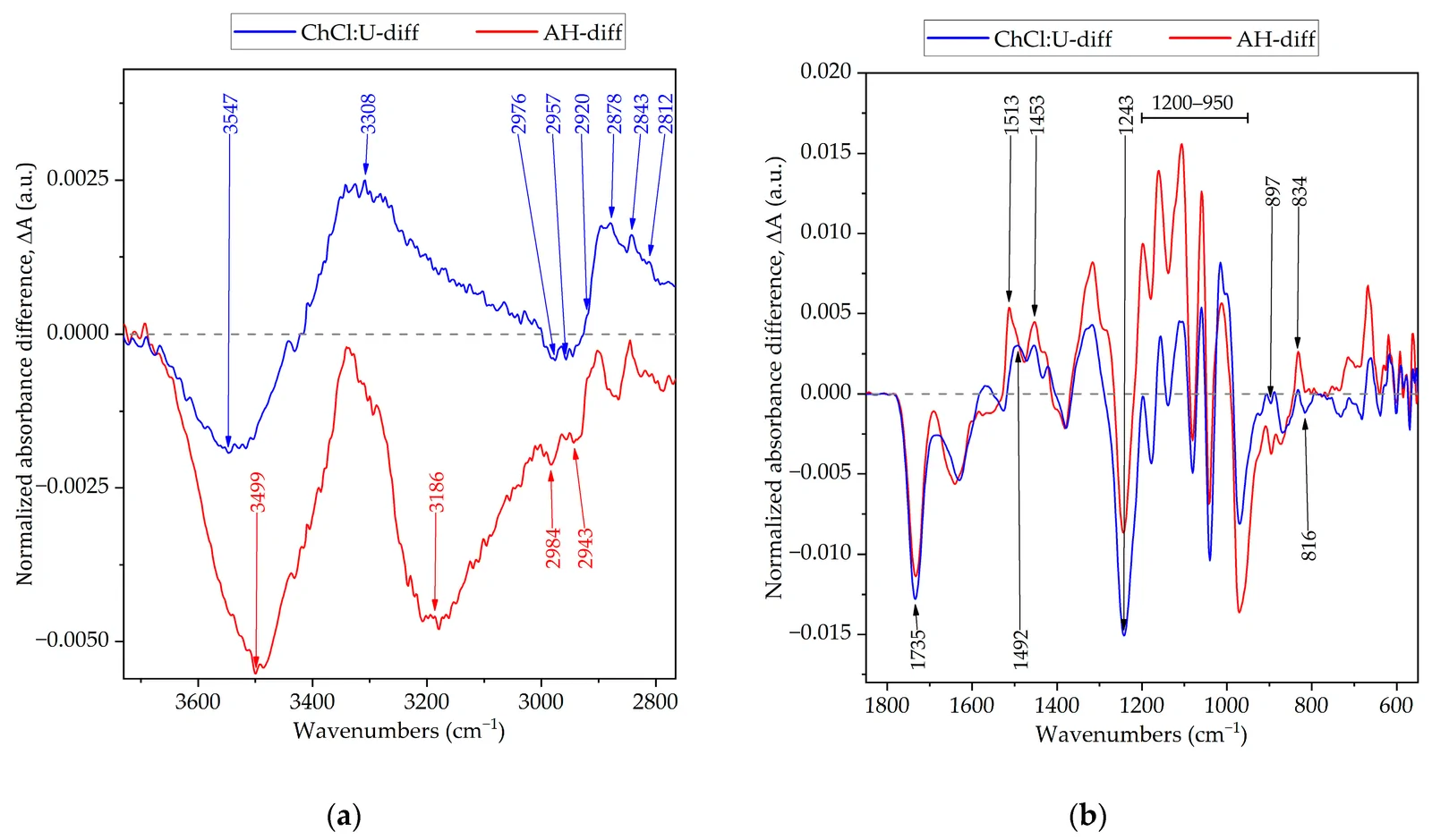
Difference spectra calculated by subtracting the Raw CC spectrum from the corresponding treated-corncob spectrum: (**a**) AH CC − Raw CC and (**b**) ChCl:U CC − Raw CC in the 3700–2760 cm^−1^ and 1800–550 cm^−1^ regions. The dashed horizontal line represents ΔA = 0.

To identify the spectral changes induced by acid hydrolysis and the subsequent DES treatment, difference spectra were calculated by subtracting the average spectrum of raw corncob from the average spectrum of each treated corncob (*n* = 3). The resulting spectra are referred to as AH-diff and ChCl:U-diff for AH-raw CC, and ChCl:U-raw CC, respectively (Figure 4). Negative peaks indicate a lower relative absorbance in the treated corncob, whereas positive peaks indicate an increased relative spectral contribution compared with the raw corncob.

As the spectra were vector-normalized prior to subtraction, the magnitude of these differences represents relative changes in spectral contributions and should not be interpreted as a quantitative measure of the amount of lignin, hemicellulose, or cellulose removed. Consequently, the FTIR results are presented as qualitative evidence of structural changes and are interpreted in conjunction with the compositional analyses.

Both difference spectra exhibited predominantly negative features in the 3700–2800 cm^−1^ region (Figure 4a). In contrast, the 1800–800 cm^−1^ region exhibited positive and negative signals. The former contains ν(O—H) from cellulose, hemicellulose, lignin, and adsorbed water; thus, the band position and shape are strongly influenced by intra- and intermolecular hydrogen bonds.

In the AH-diff, the broad band at ~3499 cm^−1^ indicates a lower contribution from free or weakly hydrogen-bonded hydroxyl groups, mainly associated with adsorbed water. The second broad negative band at ~3186 cm^−1^ reflects changes in more strongly hydrogen-bonded OH groups. These changes are consistent with the removal of hydroxyl-rich hemicelluloses, particularly arabinoxylans, during hydrolysis. Moreover, acid treatment can disrupt hydrogen bonds among hemicellulose, cellulose, and lignin [53]. Overall, this spectrum provides evidence of dehydration and reorganization of the hydrogen-bonding network after acid hydrolysis.

The ChCl:U-diff spectrum showed a negative broad signal above ~3547 cm^−1^ and a positive band near 3308 cm^−1^, indicating a displacement of the O-H envelope towards lower wavenumbers. This means that weakly hydrogen-bonded hydroxyl groups decreased, whereas the relative contribution of more strongly hydrogen-bonded hydroxyl groups increased. Removal of lignin and residual hemicellulose exposed the cellulose-rich fraction of the samples and altered the interaction among cellulose chains; hydroxyl groups previously involved in lignin–carbohydrate interactions could then reorganize into stronger cellulose–cellulose hydrogen bonds. Residual urea could also contribute at the N—H stretching region, 3250–3400 cm^−1^, but the broad shape of the band and its negative-positive profile suggest a shift in the cellulose O—H envelope.

The 3000–2800 cm^−1^ region includes asymmetric and symmetric stretching vibrations of CH_3_ and CH_2_ groups; these vibrations occur in lignin side chains, hemicellulose acetyl substituents, extractives, and polysaccharides. The AH-diff spectrum remained negative throughout this region, with marked features near 2984 and 2943 cm^−1^. The decrease in CH_3_-related absorbance supports the removal of acetyl substituents from xylans. This is further supported by the concurrent decrease near the 1735 and 1243 cm^−1^ bands, which include contributions from acetylated hemicellulose. Extractives’ removal and partial loss of lignin side chains may also contribute to this.

Negative signals around 2976–2957 cm^−1^ and positive bands from ~2920 to 2812 cm^−1^ are observed in the ChCl:U-diff spectrum. This pattern agrees with a lower relative contribution from methyl-rich structures and a greater relative contribution from methylene-rich structures. The positive CH_2_ signals reflect the increased relative abundance of the cellulose-rich fraction. These spectral changes reflect the increased proportional contribution of cellulose CH and CH_2_ after the removal of hemicellulose and lignin.

Regarding the ν(C=O) vibrations, both difference spectra showed a strong negative band at 1735 cm^−1^ (Figure 4b). The difference values were approximately −0.0113 for AH-diff and −0.0128 for ChCl:U-diff. The more negative signal in ChCl:U suggests additional removal of esterified carbonyl structures. While most hemicellulose removal occurred during acid hydrolysis, the ChCl:U treatment likely removed part of the residual acetylated hemicellulose, ester-bound hydroxycinnamates, and lignin-carbohydrate complexes.

The positive peaks near 1513 cm^−1^ for both difference spectra had values of +0.00537, and +0.00301 for AH-diff, and ChCl:U-diff, respectively. The stronger positive signal in AH-diff reflects the increased vibrational contribution of lignin following hemicellulose removal. On the contrary, in ChCl:U-diff, the signal weakened and shifted toward ~1492 cm^−1^, indicating that part of the lignin exposed after acid hydrolysis was subsequently removed. A similar trend was observed at 1453 cm^−1^, where the positive contributions in both spectra indicate an increased relative abundance of cellulose CH_2_ groups after removal of hemicellulose and lignin.

Among the lignin-associated regions, the largest difference between treatments occurred at 1243 cm^−1^. This band arises from ν(C—O) from guaiacyl lignin units and aryl—O structures (Table S4); acetylated hemicellulose, especially xylans (in the corncob context), also contributes strongly. Thus, the stronger negative signal after ChCl:U treatment suggests simultaneous loss of lignin and hemicellulose.

The 1200–950 cm^−1^ region showed alternating positive and negative signals in both difference spectra. AH-diff showed positive peaks at 1160, 1106, and 1060 cm^−1^ and negative peaks at 1040 and 971 cm^−1^. ChCl:U-diff showed negative peaks at 1177, 1081, 1040, and 971 cm^−1^ and positive peaks at 1156, 1110, 1060, and 1015 cm^−1^. None of these bands are associated with lignin molecular vibrations.

The negative peak at 895 cm^−1^ is within the region assigned to C—H deformation of β-glycosidic linkages in cellulose and other β-linked polysaccharides. This negative signal does not necessarily indicate extensive cellulose loss, but it may arise from the removal of β-linked hemicellulose or the reorganization of amorphous cellulose.

Bands in the 850–810 cm^−1^ region are commonly associated with out-of-plane aromatic C—H deformation of substituted lignin rings, including syringyl-related structures. Some polysaccharide vibrations may also contribute. The AH-diff spectrum showed a positive peak at 834 cm^−1^, which agrees with the relative enrichment and greater exposure of residual lignin after hemicellulose removal. The corresponding signal in ChCl:U-diff was almost completely suppressed, with a value of +0.00021. A negative contribution also appeared near 816 cm^−1^ after ChCl:U treatment. The decrement in the 834 cm^−1^ positive signal after ChCl:U treatment indicates removal or modification of these substituted aromatic lignin structures. The negative contribution near 816 cm^−1^ reinforces this interpretation.

The trends observed in the difference spectra were supported by the integration of the selected regions in the Raw CC, AH CC, and ChCl:U CC spectra (Figure S3). The lignin-associated areas of 1575–1614, 1488–1513, and 793–851 cm^−1^ regions increased after acid hydrolysis. Conversely, ChCl:U treatment reduced the areas of the same regions, reflecting partial delignification. The greatest decrease occurred in the 1212–1292 cm^−1^ region, as expected, because this region contains overlapping contributions of lignin and hemicellulose, with hemicellulose contributing more strongly to the observed changes. Thus, the reduction in the integrated area in the 1212–1292 cm^−1^ region after acid treatment indicates mostly the removal of hemicellulose. In contrast, the additional decrease following DES treatment suggests both residual hemicellulose and partial lignin removal.

### 2.5. Corncob Surface Morphology and Elemental Composition

**Figure 5:**
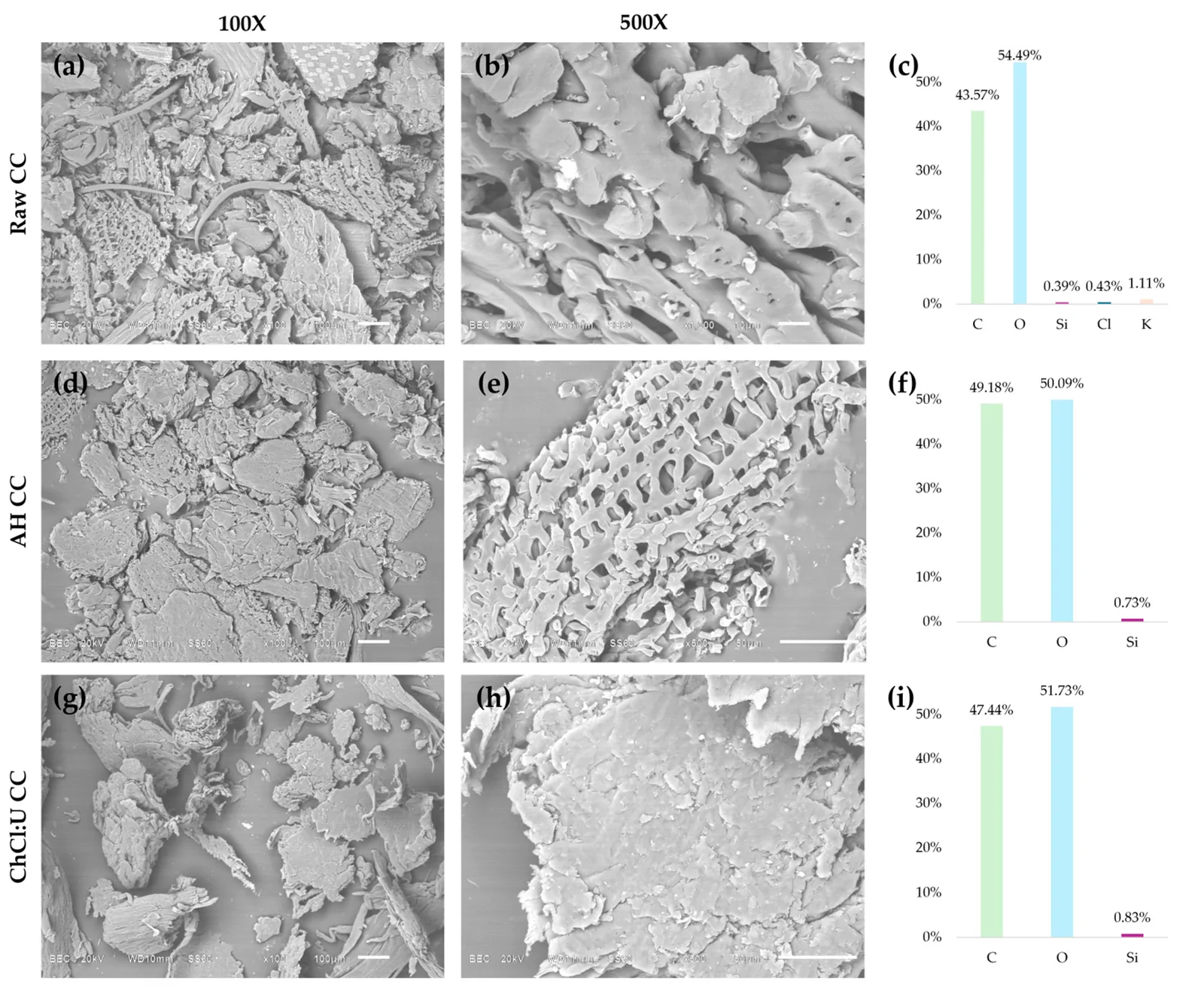
SEM micrographs and corresponding SEM-EDS elemental composition of (**a**–**c**) Raw CC, (**d**–**f**) AH CC, and (**g**–**i**) ChCl:U).

The corncob is the hard, cylindrical core that holds the corn kernels. Its macrostructure consists of three concentric layers: the outer glume, the woody ring, and the pith, each with distinct physical properties and densities, resulting in a heterogeneous material [36,54]. SEM micrographs of the untreated biomass revealed a compact and continuous surface; the cell-wall architecture remained intact, and the vascular bundles were clearly distinguishable. Structures observed, such as what appears to be glandular hairs and the hollow tubular structures shown in Figure 5a,b, correspond to the glume and woody ring, respectively [55].

Micrographs also revealed progressive disruption of the corncob structure after sulfuric acid and ChCl:U pretreatments. Sulfuric acid pretreatment generated surface erosion and exposed fibers (Figure 5d,e). At 500× magnification, the cell walls exhibited partial collapse, consistent with that observed by Liu et al. [56] after treating corncob using sulfuric acid. The subsequent ChCl:U treatment produced extensive fissures; the surface appears highly fractured, with extensive delamination and fragmentation.

To further investigate the surface characteristics of the biomass, SEM-EDS analysis was performed on the untreated (Raw CC), sulfuric acid-treated (AH CC), and ChCl:U-treated samples. The analysis indicated that oxygen (54.49%) and carbon (43.57%) were predominant elements in the raw corncob (Figure 5c); minor amounts of potassium (1.11%), chlorine (0.43%), and silicon (0.39%) were also detected. Potassium was the most abundant inorganic element; this is consistent with previous reports on biomasses [57]. Moreover, silicon is commonly accumulated in the aboveground tissues of graminaceous plants such as corn, where it can account for up to 2% of the dry weight and contributes to mechanical strength alongside lignin [36,58]. In lignocellulosic biomass, chlorine originates from chloride ions absorbed by plant roots from the soil solution and is commonly present as water-soluble inorganic salts associated with potassium or sodium [59].

Although the elemental composition was comparable to previously reported values (Table 2), slight differences in the relative abundance of elements were observed. These variations can be attributed to differences in corn cultivar, geographical region, soil composition, agronomic and harvesting practices, and the analytical techniques employed [60].

Regarding the pretreatments, carbon and oxygen remained the predominant elements in all samples, although slight changes in their relative abundance were observed after pretreatment. Representative EDS spectra of the samples are provided in the Supplementary Materials (Figure S4).

The decrease in the relative oxygen content after acid hydrolysis (from 54.5% to 50.1%) may be attributed to the elimination of hemicellulose, which is an oxygen-rich compound, and to the dehydration of the sample during the treatment. Potassium, nitrogen, and chlorine, present in the untreated corncob, were no longer detected after sulfuric acid pretreatment (Figure S4f). This finding suggests that these elements, present primarily as water-soluble inorganic salts or free ions, were leached into the acidic hydrolysate during pretreatment and removed during the subsequent washing steps. After the ChCl:U treatment, carbon and oxygen contents were similar to those of AH samples, suggesting that delignification primarily modified biomass microstructure rather than its overall elemental composition.

## 3. Materials and Methods

### 3.1. Materials

Corncob was collected from agricultural fields of Guasave, Sinaloa (25°33′56″ N 108°28′19″ O), in the northwest region of Mexico. Biomass was milled and sieved to obtain a 1 mm particle size (No. 18 test sieve in accordance with ASTM E11-24 [64]). Prior to DES treatment, corncob was treated with diluted sulfuric acid (1.36% *v*/*v*, 45 min, 121 °C) to remove hemicellulose. The pretreated corncob was washed with distilled water until a neutral pH was reached and then oven-dried at 50 °C overnight. Choline chloride, urea, glycerol, and all the reagents were of standard analytical grade with purities above 98% and used as received.

### 3.2. Structural Compositional Analysis

Biomass compositional analysis was performed following NREL protocols [65]. Biomass was subjected to hydrolysis with concentrated sulfuric acid (72% *v*/*v*) for 60 min; subsequently, the sample was diluted to an acid concentration of 4% *v*/*v* and subjected to hydrolysis at 121 °C for one hour in an autoclave. The solid residue was recovered via vacuum filtration, dried at 105 °C for 24 h and then calcinated at 575 °C for 24 h. Cellulose and hemicellulose content was calculated by the measurement of monomeric sugars on the liquid fraction by UPLC using an Ion exclusion column (HPX-87H, BIO-RAD, Hercules, CA, USA) at 50 °C, a refraction index detector, and H_2_SO_4_ 5 mM as the mobile phase at a 0.6 mL/min flux; samples were treated with a barium oxide and zinc sulfate solution and filtered using a 0.45 um nylon filter [66]. Lignin, ash, and moisture content were calculated gravimetrically. All assays were performed in triplicate.

### 3.3. Synthesis of DES and Lignin Removal from Corncobs

Choline chloride (HBA) was mixed with urea or glycerol as HBDs at a molar ratio of 1:2 (HBA:HBD). Components were mixed at 80 °C with continuous stirring (300 rpm) until a clear, homogeneous solution was obtained [67].

DESs were added to corncob in a solid–liquid ratio of 1:10. Treatments were performed in a sand bath at a constant temperature. To assess the effect of temperature on lignin removal, three temperatures were analyzed (80 °C, 100 °C, and 120 °C) for 2 h each. The influence of time was evaluated by performing tests of 2, 4, and 6 h at 100 °C. A one-way analysis of variance (ANOVA) was performed for each treatment, followed by Tukey’s multiple comparison test to determine significant differences among the means with a confidence level of 95% using Minitab Statistical Software version 21.1.0 (Minitab, LLC., State College, Pennsylvania, PA, USA).

After the treatments, reactions were stopped by the addition of water. Samples were filtered and methanol was added as an antisolvent. This mixture was kept at 200 rpm for an hour. Treated corncob was filtered and washed until a colorless effluent was obtained; after washing, samples were dried overnight at room temperature. Lignin removal percentage was calculated using Equation (2).

(2) $$Lignin\;removal\;\left(\%\right)=\left[\left(1-\left(\left(\frac{L_{pt}}{L_{ah}}\right)\times \frac{B_{pt}}{B_{in}}\right)\right)\right]$$

where:

*L_ah_* = lignin in biomass after acid hydrolysis;

*L_pt_* = lignin in biomass after DES pretreatment;

*B_in_* = biomass before DES pretreatment;

*B_pt_* = biomass after DES pretreatment.

### 3.4. Box–Behnken Design for Lignin Removal Optimization

A Box–Behnken Design (BBD) was employed to optimize lignin removal from corncob using deep eutectic solvents. The independent variables evaluated were temperature (A), time (B) and the solid-to-liquid ratio (C). The percentage of lignin removal (%) was considered the response variable. The variables were evaluated at three levels (−1, 0 and +1). A total of 15 experimental runs, including three replicates at the center point were performed. The experimental design matrix was generated using Design-Expert software version 13.0 (STAT-EASE Inc., Minneapolis, MN, USA), as shown in Table 3.

### 3.5. Statistical Analysis

The statistical analysis of the experimental data was performed using an analysis of variance (ANOVA). The significance of the model and individual terms was assessed using an F-test, with a significance level of 0.05. Model adequacy was evaluated using the lack-of-fit test, and by examining R-squared and adjusted R-squared statistics to evaluate the quality of the model fit.

### 3.6. FTIR Biomass Analysis

Three independent batches of each treatment, Raw CC, AH CC and ChCl:U CC, were analyzed by infrared spectroscopy using a Bruker Tensor 27 spectrometer equipped with a DLaTGS detector (Billerica, MA, USA). A Pike Technologies MIRacl ATR with a ZnSe crystal (Madison, WI, USA) and its high-pressure clamp was employed. The clean ATR crystal was used for background measurements and was obtained by co-adding 25 scans; in turn, each sample spectrum was acquired by co-adding 60 scans. All measurements were performed in the mid-IR region at a 4 cm^−1^ resolution. To improve the spectral quality by minimizing variations associated with sample particle size and ensuring uniform contact between the samples and the ATR crystal under applied pressure, all samples were sieved (ASTM standard mesh 18) to a uniform particle-size range prior to FTIR analysis.

The spectral analysis was performed using Quasar version 1.13.1 [68]. Spectral pre-processing was performed in the following order: atmospheric correction to minimize water and CO_2_ contributions, baseline correction, and vector (L2) normalization [69]. Subsequently, the spectra obtained for each treatment were averaged, and the lignin-associated absorption bands within the 793.0–850.7 cm^−1^, 1438–1531 cm^−1^, 1574.8–1614.2 cm^−1^, and 1213.6–1291.7 cm^−1^ regions were integrated for further analysis.

### 3.7. Microstructure and Elemental Composition

For SEM, samples were covered with a thin gold film to improve conductance using a Denton Vacuum Desk V sputter coater (Moorestown, NJ, USA); surface changes were observed using a JEOL JSM-6610 Scanning Electron Microscope (Tokyo, Japan).

## 4. Conclusions

Deep eutectic solvents are well known for their ability to remove lignin from the lignocellulosic matrix, thereby making the cellulose and hemicellulose polymers more available for further enzymatic digestion. Choline-chloride-based DESs are widely used due to their low cost and toxicity compared to other solvents. This study evaluated two ChCl-based DESs in the corncob delignification process; under the conditions evaluated, ChCl:U exhibited a higher degree of delignification compared to the ChCl:Gly system. Through the response surface methodology, we found that reaction time is the variable with the greatest effect on lignin removal when urea is applied as a HBD, followed by temperature; the solid–liquid ratio had no significant effect on delignification of corncob.

ChCl:U removed ~60% of the initial lignin in biomass, as corroborated by FTIR and elemental composition; lignin-related signals were reduced, while glucan spectral contributions increased. These results suggest that ChCl:urea effectively removed lignin, allowing for cellulose recovery and subsequent saccharification.

## Figures and Tables

**Table 1:** ANOVA for quadratic model of lignin removal using ChCl:U.

Source	Sum of Squares	Df	Mean Square	F-Value	*p*-Value	
Model	751.11	9	83.46	5.14	0.0430	significant
A-Temperature	191.80	1	191.80	11.82	0.0185	
B-Time	214.68	1	214.68	13.23	0.0150	
C-SLR	50.47	1	50.47	3.11	0.1381	
AB	58.41	1	58.41	3.60	0.1163	
AC	4.01	1	4.01	0.2473	0.6401	
BC	6.62	1	6.62	0.4078	0.5512	
A^2^	30.61	1	30.61	1.89	0.2281	
B^2^	150.17	1	150.17	9.25	0.0287	
C^2^	73.30	1	73.30	4.52	0.0869	
Residual	81.16	5	16.23			
Lack of fit	68.41	3	22.80	3.58	0.2261	not significant
Pure Error	12.75	2	6.37			
Cor Total	832.27	14				

**Table 2:** Corncob elemental composition and comparison with literature.

Element (%)	C	O	Si	Cl	K	H	N	S	Al	Na	Ca	Mg	Fe	Technique	Reference
	43.57	54.49	0.39	0.43	1.11	-	-	-	-	-	-	-	-	SEM-EDS	This work
	48.00	43.00	0.66	0.25	0.83	6.04	0.77	0.13	0.05	0.02	0.06	0.051	0.03	CHNS	[60]
	44.40	48.27	0.44	-	1.53	5.60	0.43	1.30	0.31	1.32	0.11	0.42	0.06	CHNS	[61]
	43.75	43.69	-	-	-	6.38	0.33	0.06	-	-	-	-	-	CHNS	[62]
	-	77.52	10.06	-	2.20	-	-	-	4.44	1.14	2.09	1.49	1.06	SEM-EDS	[63]

**Table 3:** Box–Behnken design matrix for lignin removal using ChCl:U.

Study Variables	Levels and Values		
	−1	0	+1
A: Temperature (°C)	80	100	120
B: Time (h)	2	5	8
C: SLR (g:mL)	1:10	1:15	1:20

## Data Availability

The raw data supporting the conclusions of this article will be made available by the authors on request.

## Supplementary Materials

The following supporting information can be downloaded at: https://www.mdpi.com/article/10.3390/molecules31183202/s1, Figure S1. ChCl:Gly lignin removal surface response graphs. (a) Interaction between reaction temperature and SLR. (b) Interaction between reaction temperature and time. (c) Interaction between reaction time and RSL; Figure S2. Externally studentized residuals versus predicted lignin removal values for the ChCl:U optimization model, used to evaluate model adequacy and homogeneity of variance; Figure S3. Box-plot of integrated normalized band areas for the 1574.8–1614.2, 1488.0–1531.0, 1213.6–1291.7, and 793.0–850.7 cm^−1^ regions; Figure S4. SEM micrographs and EDS spectra of (a,b) raw corncob, (c,d) sulfuric acid-treated corncob, and (e,f) ChCl:U-treated corncob; Table S1. Composition and solid recovery of corncob at different stages of the pretreatment process; Table S2. ANOVA for quadratic model of lignin removal using ChCl:Gly; Table S3: Box–Behnken experimental design matrix for lignin removal using ChCl:U; Table S4: FTIR band assignments for raw, acid-hydrolyzed, and ChCl:U-treated corncob samples.

## Abbreviations

The following abbreviations are used in this manuscript:

DESs	Deep Eutectic Solvents
CC	Corncob
ChCl	Choline Chloride
SLR	Solid–Liquid Ratio
LB	Lignocellulosic Biomass
HBD	Hydrogen Bond Donor
HBA	Hydrogen Bond Acceptor
FTIR	Fourier Transform Infrared Spectroscopy
SEM	Scanning Electron Microscopy
U	Urea
