# Supplementary material for: Towards Efficient Lignin Removal: A Box–Behnken Design Approach to Corncob Delignification Using Deep Eutectic Solvents
Source: Molecules. 2026 Sep 11;31(18):3202. doi: 10.3390/molecules31183202 (PMC13609433; doi:10.3390/molecules31183202)
Supplement: Supplementary file 1 [file molecules-31-03202-s001.zip › molecules-4541562-supplementary.pdf]

Supplementary Materials

Table S1. Composition and solid recovery of corncob at different stages of the pretreatment process

Biomass	Initial dry biomass	Solid recovery	Glucans	Xylans	Lignin
Raw corncob	-	-	34.59 ± 2.56	33.60 ± 1.94	20.19 ± 0.133
After acid hydrolysis (AH CC)	20 g	65 %	-	-	30.59 ± 0.912
After DES treatment (ChCl:U CC)	15 g	70 %	65.37 ± 0.82	1.74 ± 0.27	24.20 ± 0.95

Table S2. ANOVA for quadratic model of lignin removal using ChCl: Gly

Source	Sum of Squares	df	Mean Square	F-value	p-value	
Model	330.91	3	110.30	2.63	0.1025	not significant
A-Temperature	293.12	1	293.12	6.99	0.0228	
B-Time	20.15	1	20.15	0.4805	0.5026	
C-RSL	17.63	1	17.63	0.4202	0.5301	
Residual	461.40	11	41.95			
Lack of Fit	459.05	9	51.01	43.44	0.0227	significant
Pure Error	2.35	2	1.17			
Cor Total	792.30	14				

Table S3: Box-Behnken experimental design matrix for lignin removal using ChCl: U

Run	Factor 1 A:Temperature °C	Factor 2 B:Time h	Factor 3 C:SLR g:mL	Response 1 Lignin removal %
**1**	100	8	20	51.836
**2**	80	5	10	38.068
**3**	100	5	15	55.867
**4**	100	2	10	41.287
**5**	120	5	20	57.475
**6**	80	5	20	50.778
**7**	100	2	20	38.055
**8**	120	2	15	51.359

**9**	120	5	10	48.772
**10**	100	8	10	49.922
**11**	80	2	15	32.831
**12**	100	5	15	53.712
**13**	80	8	15	49.986
**14**	120	8	15	53.229
**15**	100	5	15	58.744

Table S4: FTIR band assignments for raw, acid-hydrolyzed, and ChCl: U treated corncob samples.

Experimental band (cm^{-1})	Proposed vibrational assignment	Main structural contribution	Reference
**1729**	ν(C=O) stretching of ester carbonyls, with possible contributions from protonated carboxylic acids and conjugated ester groups	Mainly acetylated hemicellulose; secondary contributions from ferulate and p-coumarate esters in arabinoxylan–lignin linkages and, to a lesser extent, uronic acids such as 4-O-methyl-D-glucuronic acid	[35,36]
**1634**	δ(H–O–H) bending of adsorbed water, aromatic skeletal ν(C=C) vibrations, and ν(C=O) of conjugated carbonyl groups	Composite band dominated by adsorbed water and lignin-associated aromatic/conjugated structures; not suitable as an isolated delignification marker	[37–41]
**1603**	Aromatic skeletal ν(C=C) stretching	Lignin aromatic rings, including p-hydroxyphenyl, guaiacyl, and syringyl units	[36,42]
**1513**	Aromatic skeletal vibrations, predominantly aromatic ring ν(C=C) modes	Lignin, particularly guaiacyl–syringyl aromatic structures	[42]
**1424**	Aromatic skeletal vibrations and C–H bending of lignin, overlapped with δ(CH_2) bending of cellulose	Mixed lignin and polysaccharide contribution; strongly associated with cellulose organization and general cell-wall modification	[38,43–45]
**1370**	δ(CH) and δ(CH_2) deformation modes of glucopyranose units; possible minor methyl and methoxy deformations	Predominantly cellulose and hemicellulose, with minor lignin contributions	[37,46]
**1320**	CH_2 wagging of cellulose, overlapped with aromatic ν(C–O) vibrations	Mixed cellulose and lignin contribution; lignin component associated with syringyl structures and condensed guaiacyl units	[37,47]
**1243**	Aromatic ν(C–O) stretching, including aryl–ether and methoxy-related vibrations;	Predominantly lignin, with contributions from guaiacyl-related structures and hemicellulose	[42]

	possible polysaccharide ν(C–O) contribution		
**1160**	Asymmetric ν(C–O–C) stretching of β-(1→4)-glycosidic linkages and carbohydrate skeletal vibrations	Mainly cellulose and hemicellulose; may include minor lignin contributions and is sensitive to moisture-induced changes in cellulose hydrogen bonding	[44,48]
**897**	C–H deformation at the anomeric carbon and vibrations associated with β-(1→4)-glycosidic linkages	Cellulose and hemicellulose; commonly associated with β-linked carbohydrate structures	[49]
**833**	Out-of-plane aromatic C–H deformation of substituted aromatic rings	Lignin-associated aromatic structures, including contributions from ferulate and p-coumarate moieties in grass lignin and lignin–arabinoxylan networks	[50,51]

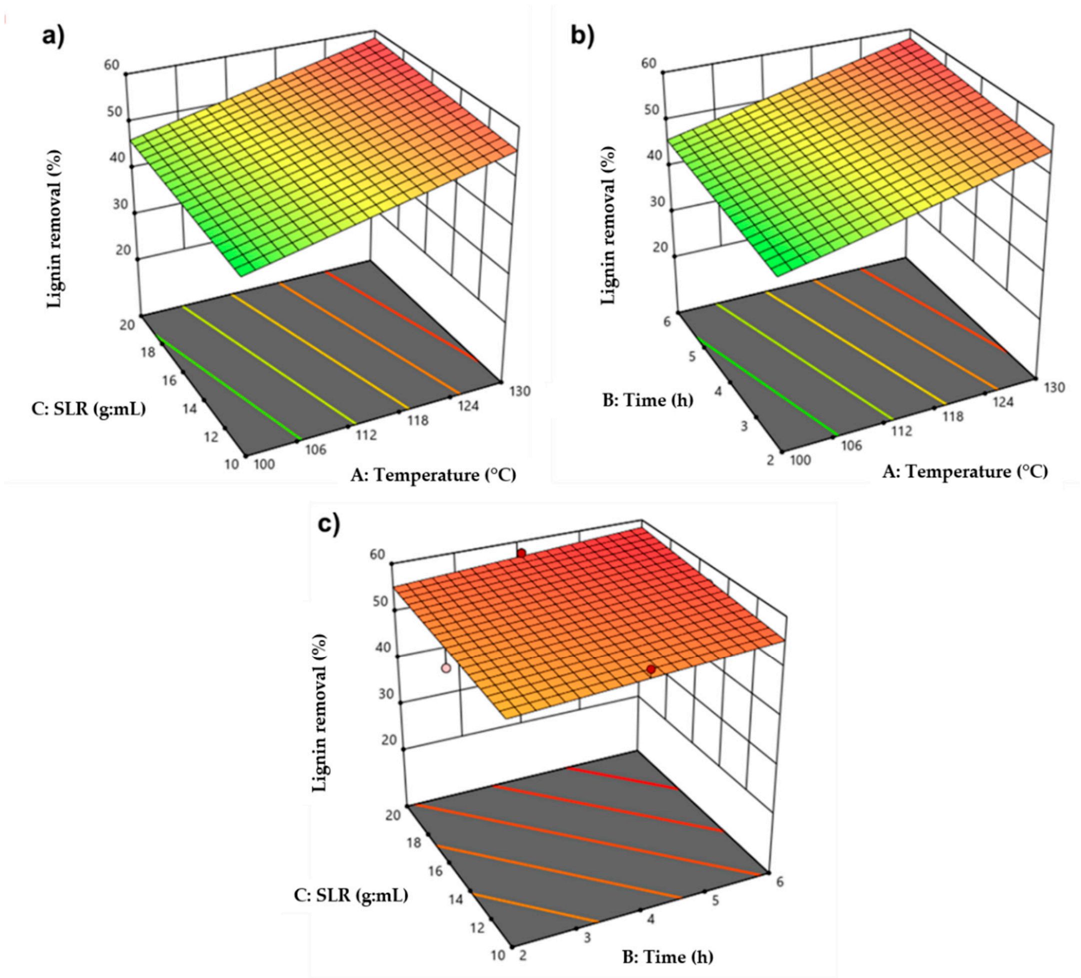

Figure S1. ChCl: Gly lignin removal surface response graphs. (a) Interaction between reaction temperature and SLR, (b) Interaction between reaction temperature and time, (c) Interaction between reaction time and RSL**.**

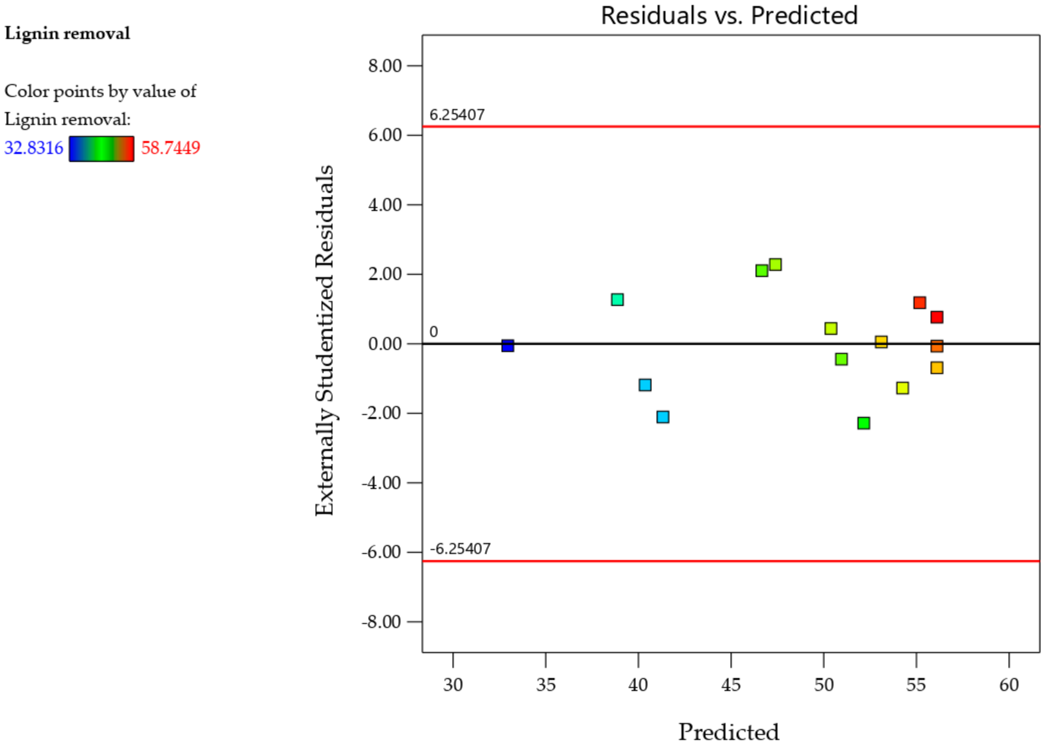

Figure S2. Externally studentized residuals versus predicted lignin removal values for the ChCl: U optimization model, used to evaluate model adequacy and homogeneity of variance.

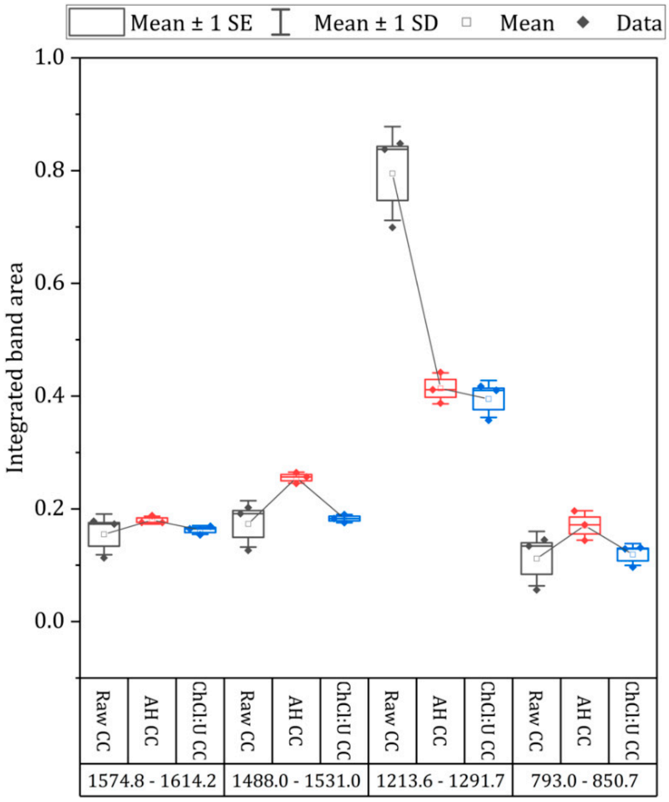

Figure S3. Box-plot of integrated normalized band areas for the 1574.8–1614.2, 1488.0–1531.0, 1213.6–1291.7, and 793.0–850.7 cm^{-1} regions.

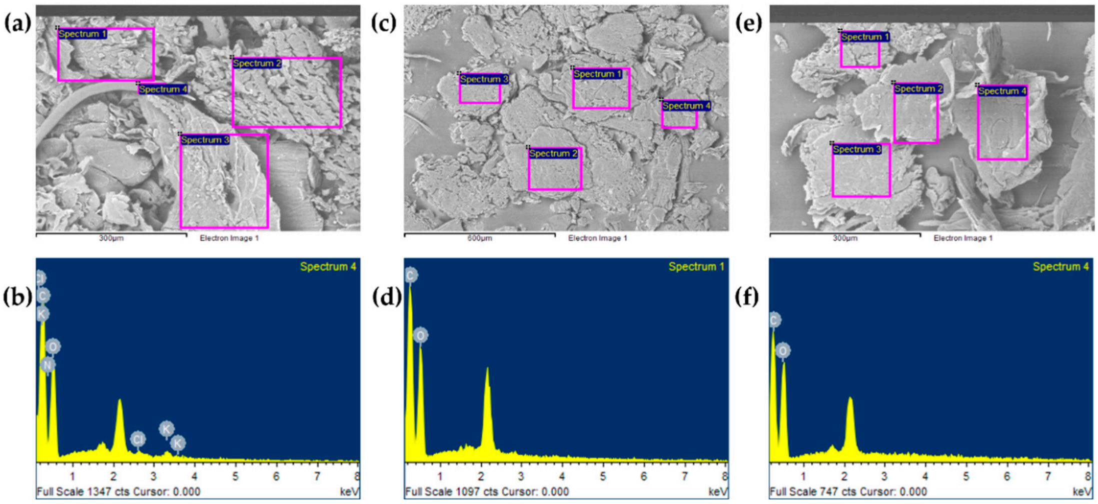

Figure S4. SEM micrographs and EDS spectra of (a-b) raw corncob, (c-d) sulfuric acid-treated corncob, and (e-f) ChCl:U-treated corncob
